# Examining Nostalgia in Old Life: Antecedence and Outcome

**DOI:** 10.1093/geroni/igaa057.1480

**Published:** 2020-12-16

**Authors:** Linming Zhou, Minjie Lu, Chaobin Zhang, Helene Fung

**Affiliations:** 1 Sun Yat-Sen University, Guangzhou, China; 2 Sun Yat-Sen University,Guangzhou, Guangzhou, China; 3 The Chinese University of Hong Kong, Sha Tin, China

## Abstract

Nostalgia is a self-conscious, bittersweet but predominantly positive and fundamentally social emotion. The regulatory model of nostalgia suggests that experiencing nostalgia can buffer against social threat (e.g. social exclusion) by providing individuals with sense of social connectedness (Sedikides, et al., 2015). In the current research, we propose that this salutary effect of nostalgia may be stronger among older adults compared to younger adults because older adults value social meaningfulness to a greater extent. Fifty-nine younger adults (Mage = 20.15, SD = 0.215) and 56 older adults (Mage = 71.02, SD = 0.679) completed daily questionnaires three times a day for ten consecutive days, and reported their emotional experience and social activities. Results showed that perceiving social threat was positively correlated with nostalgia experience reported at the subsequent time point, and this association was stronger among older adults. In addition, nostalgia positively correlated with subsequent social activities among the older participants but not among the younger participants. These findings highlight that nostalgia brings beneficial psychological (sense of social connectedness) and behavioral (social engagement) outcomes to older adults.

